# Synthesis of Iron(II)–N-Heterocyclic Carbene Complexes: Paving the Way for a New Class of Antibiotics

**DOI:** 10.3390/molecules25122917

**Published:** 2020-06-24

**Authors:** Carolina S. Vinagreiro, Rita Lopes, Beatriz Royo, Gabriela Jorge Da Silva, Mariette M. Pereira

**Affiliations:** 1 Coimbra Chemistry Centre, University of Coimbra, Rua Larga, 3004-535 Coimbra, Portugal; carolina_svinagreiro@hotmail.com; 2 ITQB NOVA, Instituto de Tecnologia Química e Biológica António Xavier, Av. da República, 2780-157 Oeiras, Portugal; rithalopes@gmail.com; 3 Faculty of Pharmacy and Center for Neurosciences and Cell Biology, University of Coimbra, Polo das Ciências da Saúde, Azinhaga de Santa Comba, 3000-548 Coimbra, Portugal; gjsilva@ci.uc.pt

**Keywords:** *N*-heterocyclic carbene, iron(II)-NHC complexes, antibacterial activity

## Abstract

The synthesis and structural modulation of five pro-ligand salts was achieved using alternative sustainable synthetic strategies, the use of microwaves being the method of choice, with an 81% yield and an E factor of 43 for **3d**. After complexation with Fe_3_(CO)_12_ by direct reaction with the appropriate pro-ligands at 130 °C, a set of iron(II) N-heterocyclic carbene (NHC) complexes were isolated and fully characterized (via ^1^H and ^13^C NMR and IR spectroscopy and elemental analysis). The antibacterial activities of the iron(II)-NHC complexes were tested against standard World Health Organization priority bacterial strains: *Staphylococcus aureus* ATCC 29213 and *Escherichia coli* ATCC 25922. The results showed a significant effect of the Fe(II)-NHC side-chain on the antibacterial activity against both Gram-negative and Gram-positive bacteria. Among all compounds, the most lipophilic iron complex, **3b**, was found to be the most active one, with a minimum inhibitory concentration of 8 µg/mL. Pioneering mechanistic studies suggested an alternative mechanism of action (OH· formation), which opens the way for the development of a new class of antibiotics.

## 1. Introduction

Antimicrobial resistance (AMR) against marketed antibiotics is one of the most serious public threats that our society currently faces [1]. Without countermeasures, projections put the number of annual global deaths caused by drug-resistant infections at nearly 10 million by 2050. The latest World Health Organization (WHO) report included an urgent alert about the dramatic phenomenon of antibiotic resistance, and prioritized a group of pathogens concerning in terms of AMR, which includes *Escherichia coli* and *Staphylococcus aureus*, among others [2]. Therefore, the development of new molecular entities or approaches capable of inactivating microorganisms without promoting drug resistance is crucial, and depends on unveiling alternative mechanisms of action for conventional drugs. Among the approaches described in the literature, the use of organometallics is considered a great challenge, since metals can not only modulate the amphiphilicity of the molecules and, consequently their interactions with membranes (uptake profile), but can also generate reactive oxygen species that can kill bacteria by alternative mechanisms. Hence, the synthesis of new organometallic compounds is an attractive approach to overcome the resistance issue, if the resulting compounds can offer a metal-specific mode of action that is not available from a purely organic parent molecule [3]. N-Heterocyclic carbene metal complexes (NHCs) have been widely explored in organometallic chemistry and homogeneous catalysis [4,5,6], and metal–NHC complexes were also recently reported as promising molecules for several medicinal applications [7,8,9]. In 2004, Youngs [10] originally reported the use of an Ag(I)-HC for antibacterial application purposes. After that, other NHC–metal complexes of silver (up to 1 µg/mL) [11], palladium (5 to 11 mm) [12], gold (up to 2 µg/mL) [13], ruthenium (6.25 to 1000 µg/mL), and rhodium (5 to >1000 µg/mL) [14] were reported to possess antibacterial activity. However, these NHC transition metal complexes are quite expensive and present remarkable cytotoxicity. The development of alternative antibacterial nontoxic NHC–metal complexes therefore remains a great challenge. Iron has arisen as a promising metal candidate due to its absence of toxicity and its redox behavior, offering a different chemical reactivity and consequently new mechanisms of drug action. Iron metal complexes are widely recognized as effective antimicrobial agents, following two main strategies: as iron(II) metal complexes linked to known antibiotics [15,16] and as modulated iron(II) metal complexes [17,18]. Regarding the first strategy, the best minimum inhibitory concentration (MIC) (0.42 µg/mL) was obtained by Xiang [16] against *Escherichia coli* using ferrocenyl-penem derivatives. In the use of Fe(II) metal complexes, a remarkable influence of the ligand structure was observed (0.27 to 512 µg/mL), an iron(II) complex based on formazan dyes being the best one both for inhibition of S. aureus and E. coli bacterial strains (0.27 µg/mL) [19].

To the best of our knowledge, the antibacterial evaluation of iron(II)–NHC complexes has never been reported. Herein, we describe the structural modulation of iron(II)–NHC complexes and their antibacterial evaluation against the WHO priority standard Gram-positive and Gram-negative bacterial strains *Staphylococcus aureus* ATCC 29213 and *Escherichia coli* ATCC 25922. MIC values up to 8 µg/mL were observed.

## 2. Results and Discussion

### 2.1. Synthesis and Characterization of Fe(II)–NHC Complexes

**Scheme 1:**
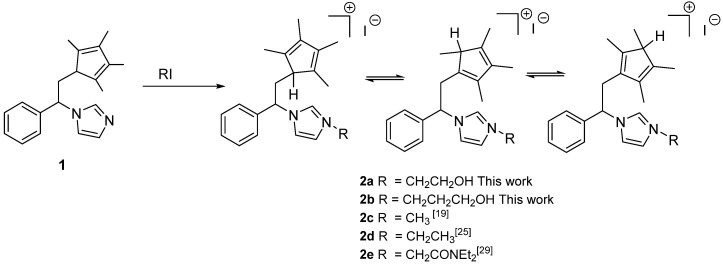
Synthesis of compounds **2a**–**e**.

Compound **1** was prepared following the procedure previously described by some of us [19]. Pro-ligands **2a** and **2b** were prepared following the same procedure as reported for the preparation of **2c**–**e** (Scheme 1). Alkylation of **1** with the appropriate alkyl iodide, RI (R = (CH_2_)_2_OH, (CH_2_)_3_OH), in acetone at 25 °C afforded the corresponding imidazolium pro-ligands **2a** and **2b**, which were isolated as yellow solids with good yields. Compounds **2a** and **2b** were fully characterized by analytical and spectroscopic methods. They were obtained as a mixture of tautomers, resulting from resonance forms of the cyclopentadiene ring [19] (Scheme 1). Alkylation of **1** with the corresponding alkyl halides was corroborated by the appearance in the ^1^H-NMR of the typical resonances at 11–9 ppm for the imidazolium proton (see Supplementary Materials).

**Figure 1:**
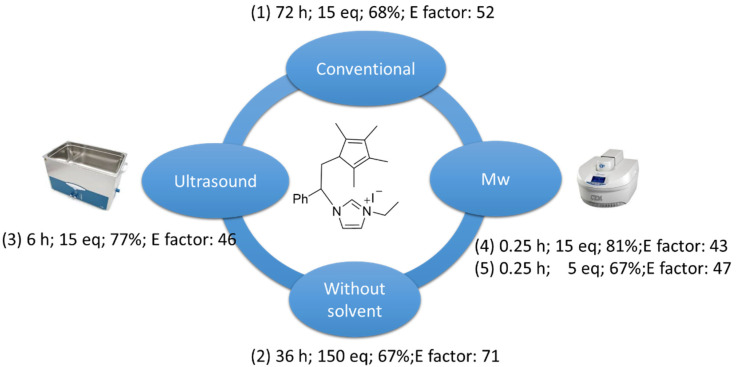
Optimization of the synthetic method using pro-ligand **2d** as a model. Abbreviations: h—hours; eq—equivalentes; E factor—environmental factor; Mw—Microwave radiation.

Our interest in finding new methodologies involving more sustainable chemical processes led us to optimize the synthetic method described above for the preparation of **2a**–**e**, by performing the reaction of **1** with RI (at the minimum quantity required to dissolve **1**) in the absence of a solvent. In this manner, the yield obtained reached similar values to that obtained by conventional methods, (Figure 1, Method 2); under ultrasound irradiation (Method 3), where a yield of 77% was obtained after 6 h of reaction (Figure 1, Method 3), or under microwave (Mw) radiation (Methods 4 and 5, P = 100 W). Interestingly, under Mw irradiation, high yields (81%) of the desired ligands were obtained in 15 min of reaction (Figure 1, Method 4). If the amount of RI was reduced to 5 eq, the yield of **2d** decreased to 67%. This strategy presented the lowest environmental impact, evaluated by E factor [20], and it was conveniently applied for the synthesis of **2c**–**e**; however, it proved to be ineffective for **2a** and **2b**, where a mixture of byproducts was obtained.

**Scheme 2:**
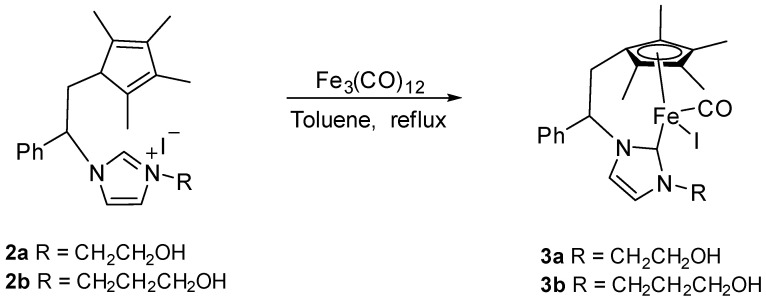
Synthesis of new iron(II)–NHC complexes **3a** and **3b**.

The new iron complexes **3a** and **3b** were conveniently prepared by direct reaction of Fe_3_(CO)_12_ with the appropriate pro-ligands **2a** and **2b**, respectively, in toluene under reflux for 16 h, following the procedure reported by some of us (Scheme 2) [21]. Complexes **3a** and **3b** were isolated as green crystalline solids in good yields (74 and 76%, respectively), and were fully characterized by IR, ^1^H, and ^13^C NMR spectroscopy, mass spectrometry, and elemental analysis.

The successful metalation was confirmed by the appearance of the characteristic carbene signal at 195 ppm in the ^13^C NMR spectra of **3a** and **3b**, in accordance with previously reported data for related half-sandwich Fe–NHC complexes (see Supplementary Materials). In addition, the ^13^C NMR spectra of **3a** and **3b** showed the characteristic resonance for the carbonyl ligand at 227 ppm. The formation of iron complexes was also corroborated by infrared spectroscopy, where compounds **3a** and **3b** showed strong carbonyl resonance at 1901 and 1900 cm^−1^, respectively.

### 2.2. Antimicrobial Activity

**Scheme 3:**
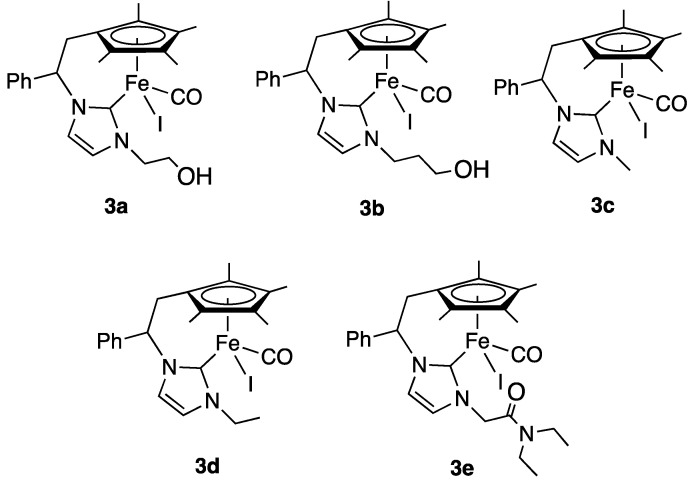
Iron(II)–NHC complexes evaluated against bacteria.

In order to evaluate the effect of NHC structure on the antibacterial activity against Gram-positive and Gram-negative bacteria, the antimicrobial efficacy of compounds **3a**–**e** (Scheme 3) was determined against two standard WHO priority strains, which were taken from the American Type Culture Collection, *Escherichia coli* (ATCC 25922) and *Staphylococcus aureus* (ATCC 29213), and the results are summarized in Table 1. The antimicrobial activity is reported in terms of the minimum inhibitory concentration (MIC, μg/mL) values, which are defined as the lowest concentration of an antimicrobial that visibly inhibits the growth of the bacteria after an overnight incubation [22].

From the analysis of the results presented in Table 1, we observed a significant effect of the Fe(II)–NHC side-chain on the antibacterial activity against both Gram-negative and Gram-positive bacteria. Regarding the MICs against S. aureus, the presence of amphiphilic hydroxyl groups gave a remarkable result and MIC values of 8 µg/mL were achieved with compounds **3a** and **3b** (Table 1, Entries 1 and 2). The same result was obtained for the complex with a hydrophobic ethyl group (**3d**) (Table 1, Entry 4). In addition, the presence of a hydrophobic methyl group (**3c**) or dimethylacetamide groups (**3e**) led to a significant decrease of the activity, by 20 and >32, respectively (Table 1, Entries 3 and 5).

Next, we evaluated the MICs of the most promising compounds against Gram-negative bacteria. A difference of just one carbon atom on the alkyl hydrophobic chain (methyl **3c** and ethyl **3d**) or on the alkyl chain of amphiphilic hydroxyl groups (ethanol **3a** and propanol **3b**) showed a strong effect on antibacterial activity, of 500 to 8 µg/mL (Table 1, Entries 3 and 4) and 62.5 to 125 µg/mL (Table 1, Entries 1 and 2), respectively.

Among the compounds tested, the most lipophilic complex (**3d**) was found to be the most active against both strains (MIC = 8 µg/mL). This side-chain effect was also observed by Özdemir [23] and Karatas [24] for Ag(I)–NHC complexes, with the most active complexes also being the most lipophilic.

Comparing the obtained results with the best iron(II) complex reported in literature [17] (0.27 µg/mL), it is possible to understand that future structural optimization will be necessary to improve the compound’s antibacterial activity. Nevertheless, this initial evaluation will pave the way for a new application of iron(II)–NHC complexes as antibacterial compounds.

### 2.3. Preliminary Mechanistic Studies

To the best of our knowledge, the biological activities of these complexes have been poorly explored to date, as they were only studied as catalysts previously [25,26]. The knowledge that metal complexes’ antibacterial activity is enhanced by reactive oxygen species (ROS) formation [27,28], coupled with our intention to apply these iron complexes as antibacterial compounds, led us to explore their capability to form ·OH radicals under biological conditions, Equation (1), using two methodologies.

(1) $$\mathrm{Fe}\left(\mathrm{II}\right)+H_{2}O_{2}\underset{}{\to }\mathrm{OH}\cdot +\mathrm{OH}-+\mathrm{Fe}(\mathrm{III})$$

**Figure 2:**
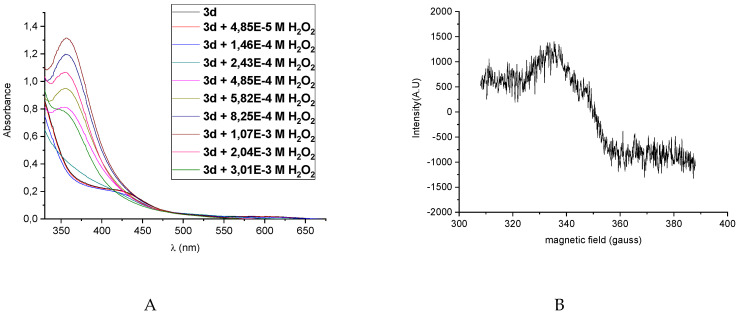

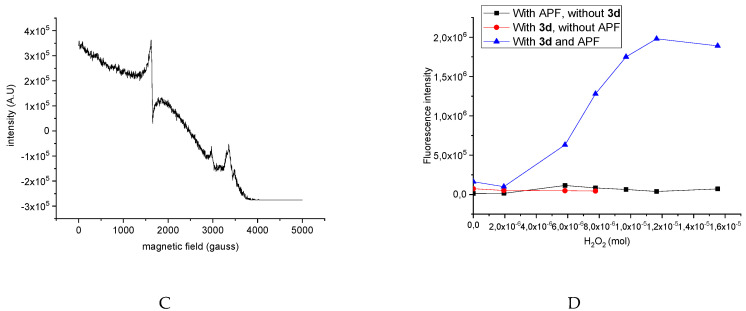
Assessment of **3d** complex oxidation: (**A**) Complex **3d** UV–Vis absorption spectra with progressive addition of H_2_O_2_; (**B**) EPR of complex **3d** at 20 °C; (**C**) EPR of complex **3d** after adding H_2_O_2_ at 20 °C; (**D**) Complex **3d** and APF fluorescence spectra upon addition of H_2_O_2_ and respective controls. Abbreviations: EPR—Electron paramagnetic resonance; APF—*p*-aminophenyl fluorescein.

1. To evaluate the metal oxidation state, UV–Vis spectra were recorded between each addition of H_2_O_2_ to a solution of complex **3d** (Figure 2A), and significant spectral changes were observed, which was attributed to iron(II) oxidation. Electron paramagnetic resonance (EPR) corroborated this evidence, since **3d** showed no signal, a typical behavior of a Fe(II) spin-down complex (Figure 2B), after which the addition of H_2_O_2_ caused the appearance of the typical signal of a Fe(III) complex (Figure 2C).
2. To detect the presence of hydroxyl radicals, a ROS indicator, *p*-aminophenyl fluorescein, was used. This probe is non-fluorescent until it reacts with the hydroxyl radical; the ROS formation could therefore be observed through the progressive detection of fluorescence upon addition of H_2_O_2_, confirming the formation of hydroxyl radicals (Figure 2D).

## 3. Materials and Methods

Compounds **1**, **2c**–**e**, and iron complexes **3c**–**e** were prepared according to previously described procedures [19,25,29].

### 3.1. Preparation of Imidazolium Pro-Ligands

*Pro-ligand***2a**. 2-Iodoethanol (0.71 mL, 9.1 mmol) was added to a solution of pro-ligand **1** (0.529, 1.8 mmol) in acetone (10 mL), and the mixture was stirred at room temperature for 4 days. The suspension was filtered and the filtrate was evaporated to dryness to yield a yellow solid, which was washed with diethyl ether and hexane and dried under vacuum. Pro-ligand **2a** was isolated as a yellow solid. Yield: 532 mg (63%). ^1^H-NMR (400 MHz, CDCl_3_): mixture of isomers: δ = 9.66–9.37 (s, N=CH-N), 7.53–6.98 (m, CH_Phenyl_, CH_Imid_), 5.65–5.39 (m. CH_linker_), 4.42–4.40 (t, N-CH_2_), 3.91–3.81 (t, CH_2_), 3.31–3.03 (m, CH_2linker_), 2.76–2.48 (m, CH_Cp*_), 1.88–1.38 (s, CH3_Cp*_), 1.15–0.86 (d, CH_3Cp*_).^13^C (^1^H) NMR (100 MHz, CDCl_3_): δ = 141.32 (N=CH-N), 136.39–120.24 (C_Phenyl_, C_Imid_), 70.48–62.41 (CH_linker_), 59.50 (CH_Cp*_), 53.82–49.31 (NCH_2_, CH_2_OH), 32.15–31.73 (CH_2Linker_), 15.40–11.95 (CH_3Cp*_). HRMS (ESI-TOF) *m*/*z* [M-I]^+^ calcd for C_22_H_29_N_2_O: 337.22800; found 337.2264 [M-I]^+^.

*Pro-ligand***2b**. 1-Iodo-3-propanol (0.71 mL, 7.4 mmol) was added to a solution of pro-ligand **1** (0.533, 1.5 mmol) in acetone (10 mL), and the mixture was stirred at room temperature for 4 days. The suspension was filtered and the filtrate was evaporated to dryness to yield a yellow solid, which was washed with diethyl ether and hexane and dried under vacuum. Pro-ligand **2b** was obtained as a yellow solid. Yield: 456 mg (65%). ^1^H NMR (400 MHz, CDCl_3_) mixture of isomers: δ = 10.08–9.69 (s, N=CH-N), 7.52–7.05 (m, CH_Phenyl_, CH_Imid_), 5.87–5.69 (m. CH_linker_), 4.55–4.44 (t, N-CH_2_), 3.62–3.18 (t, CH_2_), 2.78 (m, CH_2linker_), 2.15 (m, CH_Cp*_), 2.11–2.03 (q, CH_2_), 1.80–1.43 (s, CH_3Cp*_), 1.23–0.91 (d, CH_3Cp*_). ^13^C (^1^H) NMR (100 MHz, CDCl_3_): δ = 144.04 (N=CH-N), 133.57–120.23 (C_Phenyl_, C_Imid_), 64.96–62.48 (CH_linker_), 56.91 (CH_Cp*_), 56.91–49.60 (NCH_2_, CH_2_OH), 47.79 (CH_2_), 32.45–31.69 (CH_2Linker_), 14.20–10.76 (CH_3Cp*_). MS (ESI-TOF) *m*/*z* [M-I]^+^ calcd for C_23_H_31_N_2_O: 351.24365; found 351.24274 [M-I]^+^.

Method 2: Similar to Method 1 but without solvent and with an excess of 150 equivalents (2.57 × 10–2 mol) of iodoethane. **2d**: 67% yield after 36 h.

Method 3: Similar to Method 1, but the mixture was exposed to ultrasound. **2d**: 77% yield after 6 h.

Method 4: Similar to Method 1, but the mixture was exposed to microwave irradiation at 80 °C, with a potency of 100 W for 0.25 h. **2d**: 81% yield. **2b**: 20% yield. **2e**: 87% yield.

Method 5: Similar to Method 4, but with an excess of 5 equivalents of iodoethane. **2b**: 67% yield.

### 3.2. General Procedure for the Preparation of Iron(II)–NHC Complexes ***3a*** and ***3b***

Method 1: A mixture of the appropriate pro-ligand (Cp*-NHCR)I (R= (CH_2_)_2_OH, (CH_2_)_3_OH) (1.38 mmol) and Fe_3_(CO)_12_ (0.46 mmol) was refluxed in toluene (15 mL) for 16 h. The solution was filtered and the filtrate was evaporated to dryness to yield a green solid, which was washed with hexane to afford the corresponding iron complexes isolated as green solids.

*Complex***3a**. Yield: 74%. ^1^H NMR (400 MHz, acetone-*d*_6_) δ = 7.59–7.53 (m, 5H, CH_Ph_), 7.07 (s, 1H, CH_imid_), 6.46 (s, 1H, CH_imid_), 6.06 (m, 1H, CH_Phlinker_), 4.5–4.4 (m, 2H N-CH_2_), 3.88–3.86 (m, 2H, CH_2_) 3.0–2.92 (m, 2H, CH_2linker_) 2.39 (s, 3H, CH_3Cp*_), 1.82 (s, 3H, CH_3Cp*_), 1.7 (s, 3H, CH_3Cp*_), 0.94 (s, 3H, CH_3Cp*_). ^13^C (^1^H) NMR (100 MHz, CDCl_3_): δ = 226.8 (CO), 194.9 (C_carbene_-_Fe_), 138.8 (C_ipso-phenyl_), 129 (CH_Phenyl_), 124 (CH_imid_), 120 (CH_imid_), 104 (C_Cp*_), 91.6 (C_Cp*_), 90.4 (C_Cp*_), 84.3 (C_Cp*_), 81.2 (C_Cp*_), 69 (CH_2_-N), 67.1 (CH_Ph-linker_), 61 (CH_linker_), 52 (CH_2linker_), 21 (CH_2_), 13 (CH_3Cp*_), 11 (CH_3Cp*_),10 (CH_3Cp*_), 9.5 (CH_3Cp*_). Selected IR data (KBr): ν (CO) 1901 vs cm^−1^. Anal. Calcd for C_23_H_27_N_2_O_2_FeI (546): C: 50.57; H: 4,98; N: 5.13; Found: C: 50.35; H: 4.77; N: 5.00.

*Complex***3b**. Yield: 76%. ^1^H NMR (400 MHz, acetone-*d*_6_) δ = 7.62–7.42 (m, 5H, CH_Ph_), 7.17 (s, 1H, CH_imid_), 6.48 (s, 1H, CH_imid_), 6.07(m, 1H, CH_Phlinker_), 4.32 (m, 2H N-CH_2_), 3.59 (m, 2H, CH_2_) 3.05-2.93 (m, 2H, CH_2linker_) 2.40 (s, 3H, CH_3Cp*_), 2.04 (CH_2_) 1.81 (s, 3H, CH_3Cp*_), 1.75 (s, 3H, CH_3Cp*_), 0.95 (s, 3H, CH_3Cp*_).^13^C (^1^H) NMR (100 MHz, CDCl_3_): δ = 226.8 (CO), 195.1 (C_carbene-Fe_), 138.49 (C_ipso-phenyl_), 129 (CH_Phenyl_), 123 (CH_imid_), 121 (CH_imid_), 104.7 (C_Cp*_), 91.7 (C_Cp*_), 90.4 (C_Cp*_), 84.3 (C_Cp*_), 81.2 (C_Cp*_), 67 (CH_linker_), 58.9 (CH_2_), 48.02 (CH_2_), 34.7 (CH_2linker_), 13 (CH_3Cp*_), 10 (CH_3Cp*_), 9.6 (CH_3Cp*_), 1.4 (CH_3Cp*_). Selected IR data (KBr): ν (CO) 1900 vs cm^−1^. Anal. Calcd for C_24_H_29_N_2_O_2_FeI (560): C: 51.45; H: 5.22; N: 5.00; Found: C: 51.90; H: 5.78; N: 4.82.

### 3.3. Antimicrobial Activity

The planktonic bacterial cells (*Escherichia coli* ATCC 25922 and *Staphylococcus aureus* ATCC 29213) were cultured in Mueller–Hinton agar (MH, Sigma Aldrich) at 37 °C overnight. Cell density was adjusted to 0.5 optical density in water, which corresponds to approximately 10^7^ CFU per mL, and was diluted 20 times. Stock solutions (1 mg/mL) of the compounds in water/DMSO were prepared. The range of compound concentrations tested was 500 mg/L to 1 mg/L. Sterile plastic disposable microplates with 96 round-bottomed wells were filled with Mueller–Hinton broth (Sigma Aldrich), the compounds, and 10 µL of the bacteria solutions. Wells with only DMSO, medium, or bacteria were used as positive, sterile, and growth controls, respectively. The microplates were incubated at 37 °C for 24 h. The lowest concentration of the compound that prevented visible growth was considered to be the minimal inhibitory concentration (MIC). The experiments were repeated three times.

### 3.4. Iron Oxidation Assessment

UV–Vis experiments: A solution of **3d** in acetonitrile (0.465 mM) was titrated with 33% hydrogen peroxide solution in water. The UV–vis absorption spectra were acquired between each addition.

EPR experiments: The EPR spectra of a Fe(II) complex solution in acetonitrile (**3d**, 1mM) and of another similar solution with 200 µL of H_2_O_2_ were acquired at 20 °C.

Hydroxyl radical detection: To a solution of **3d** complex (0.465 mM) was added 12.5 μM of a solution containing *p*-aminophenyl fluorescein (APF) in a sodium phosphate buffer (0.1 M, pH 7.4). H_2_O_2_ was then progressively added, and the fluorescence was measured using excitation/emission wavelengths of 490/515 nm. The ROS formation was observed through the appearance of fluorescence. Two control experiments were also done, one without APF and other without the complex **3d**.

## 4. Conclusions

In summary, the synthesis of a set of iron(II)–NHC complexes was optimized, with the microwave method being the most sustainable. All complexes were tested against standard bacterial strains, both Gram-negative and Gram-positive (*Staphylococcus aureus* and *Escherichia coli*, respectively). The results demonstrated a strong effect of the Fe(II)–NHC side chain on the antibacterial activity for both strains. A remarkable MIC of 8 µg/mL for S. aureus and E. coli was found for the most lipophilic complex (**3d**). Additionally, the proof of concept regarding the oxidation of these complexes and potential ROS formation in biological media was successfully achieved. These results are clearly a good starting point for the development of new applications of iron(II)–NHC complexes, and for encouraging the scientific community towards the development of new compounds of this family as antibacterial compounds.

## Figures and Tables

**Table 1:** MIC values of compounds used for antibacterial test.

Entry	Compounds	R	MIC (µg/mL)	
			SA	EC
1	**3a**	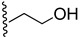	8	62.5
2	**3b**	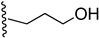	8	125
3	**3c**	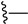	20	500
4	**3d**	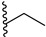	8	8
5	**3e**	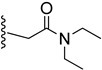	>32	n.d.

Abbreviations: MIC: minimal inhibitory concentration (μg/mL); EC: *Escherichia coli*; SA: *Staphylococcus aureus*; n.d.: not determined.

## Supplementary Materials

The following are available online at https://www.mdpi.com/1420-3049/25/12/2917/s1, Figure S1. ^1^H-NMR of **2a** in CDCl_3_.; Figure S2. ^13^C-NMR of **2a** in CDCl_3_.; Figure S3. ESI mass spectrum of **2a** acquired in positive mode.; Figure S4. ^1^H-NMR of **2b** in CDCl_3_.; Figure S5. ^13^C-NMR of **2b** in CDCl_3_.; Figure S6. ESI mass spectrum of **2b** acquired in positive mode.; Figure S7. ^1^H-NMR of **3a** in acetone-*d*_6_.; Figure S8. ^13^C-NMR of **3a** in acetone-*d*_6_.; Figure S9. Infrared spectrum (KBr) of **3a**.; Figure S10. ^1^H-NMR of **3b** in acetone-*d*_6_.; Figure S11. ^13^C-NMR of **3b** in acetone-*d*_6_.; Figure S12. Infrared spectrum (KBr) of **3b**.
