# Supplementary material for: Synthesis of Iron(II)–N-Heterocyclic Carbene Complexes: Paving the Way for a New Class of Antibiotics
Source: Molecules. 2020 Jun 24;25(12):2917. doi: 10.3390/molecules25122917 (PMC7356145; doi:10.3390/molecules25122917)
Supplement: Supplementary file 1 [file molecules-25-02917-s001.pdf]

Supplementary information

Synthesis of Iron(II)–N-Heterocyclic Carbene Complexes: Paving the Way for a New Class of Antibiotics

Carolina S. Vinagreiro [1], Rita Lopes [2], Beatriz Royo [2,*], Gabriela Jorge Da Silva [3] and Mariette M. Pereira [1,*]

[1] Coimbra Chemistry Centre, University of Coimbra, Rua Larga, 3004-535, Coimbra; carolina_svinagreiro@hotmail.com (C.S.V.)

[2] ITQB NOVA, Instituto de Tecnologia Química e Biológica António Xavier, Av. da República, 2780-157 Oeiras, Portugal; rithalopes@gmail.com (R.L.)

[3] Faculty of Pharmacy and Center for Neurosciences and Cell Biology, University of Coimbra, Polo das Ciências da Saúde, Azinhaga de Santa Comba, 3000-548 Coimbra, Portugal; gjsilva@ci.uc.pt (G.J.D.S.)

* Correspondence: mmpereira@qui.uc.pt (M.P.); broyo@itqb.unl.pt (B.R.)

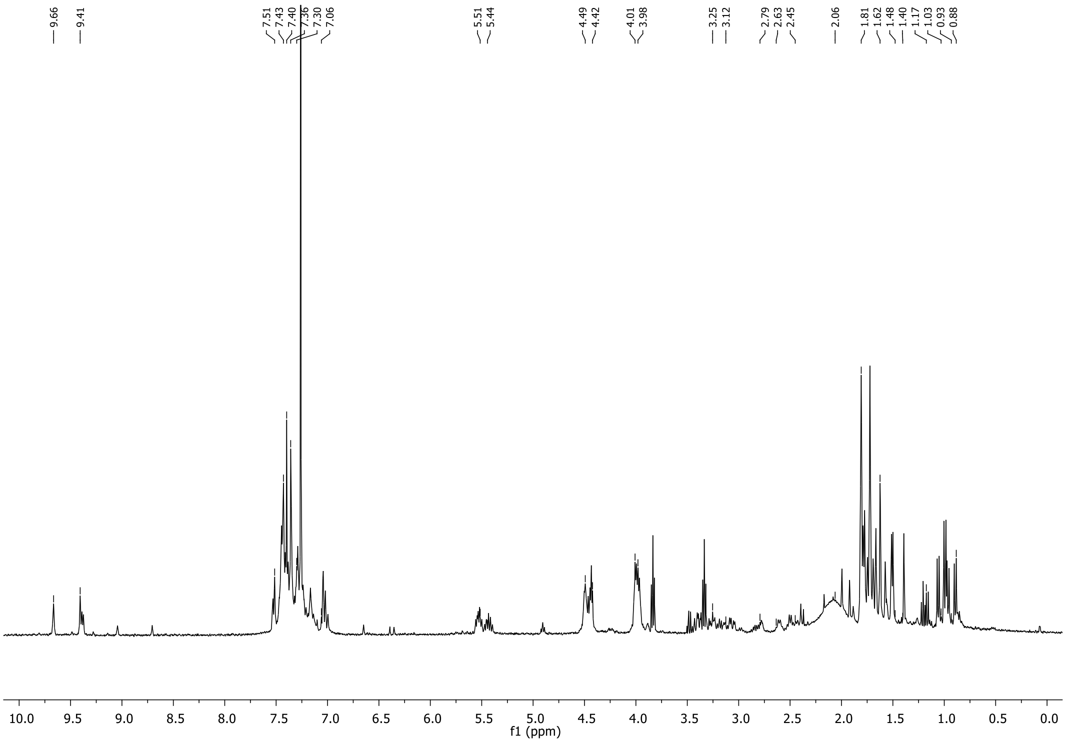

Figure S1. ^{1}H-NMR of **2a** in $CDCl_3$.

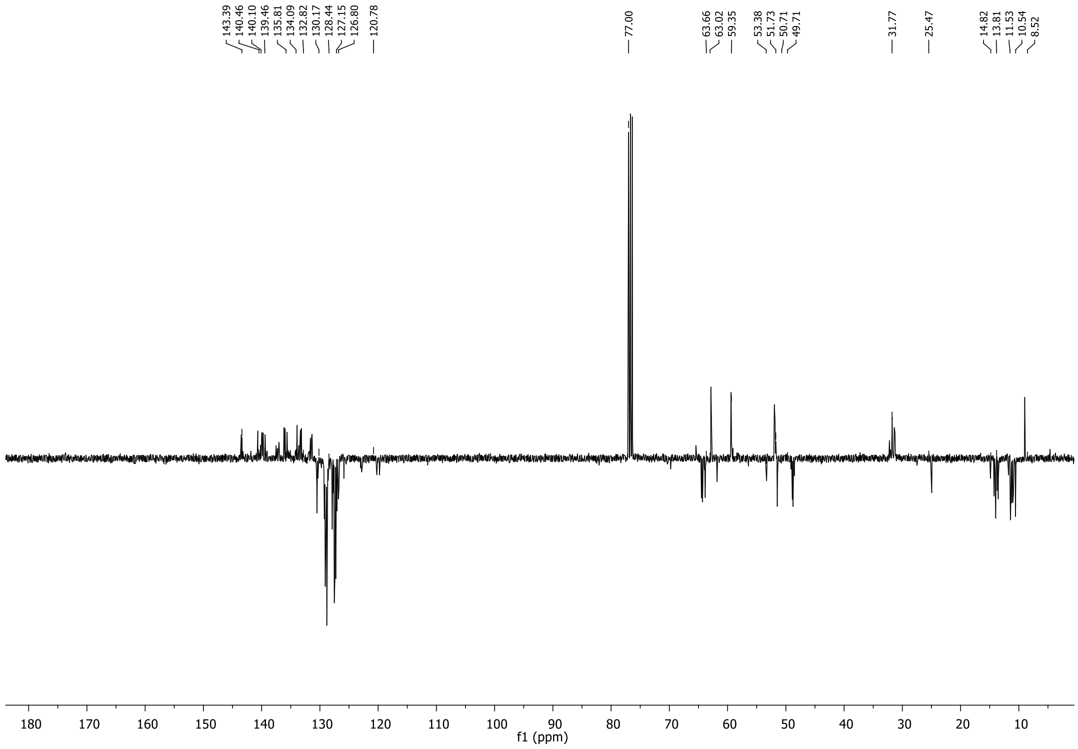

Figure S2. ^{13}C-NMR of **2a** in $CDCl_3$.

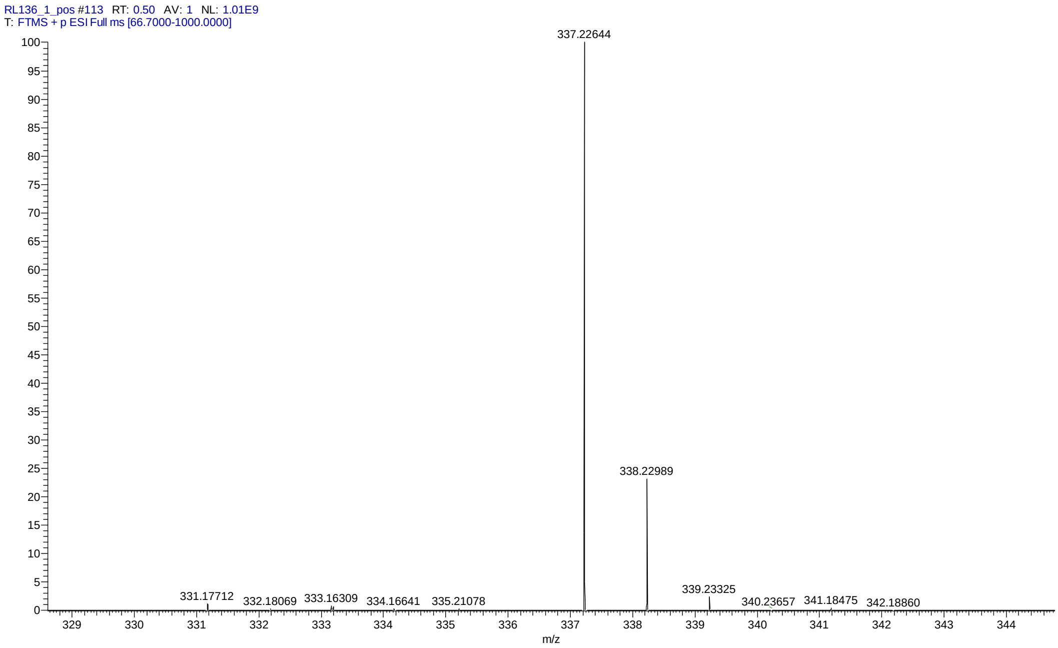

Figure S3. ESI mass spectrum of **2a** acquired in positive mode.

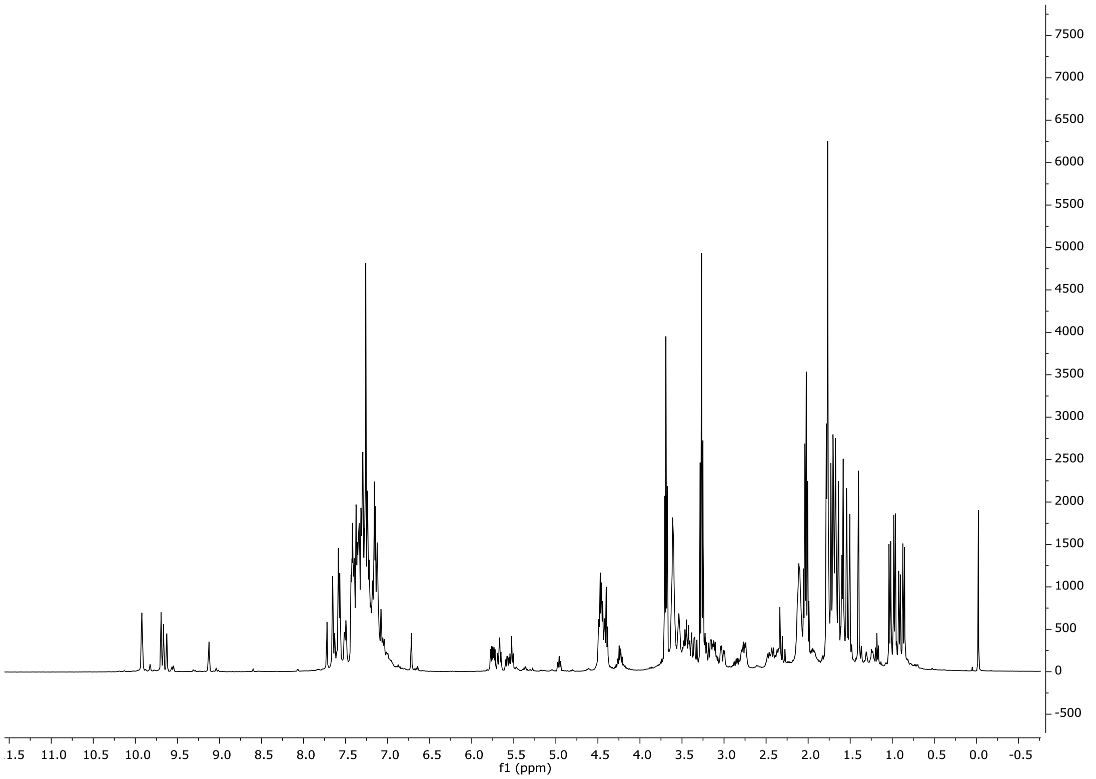

Figure S4. ^{1}H-NMR of **2b** in $CDCl_3$.

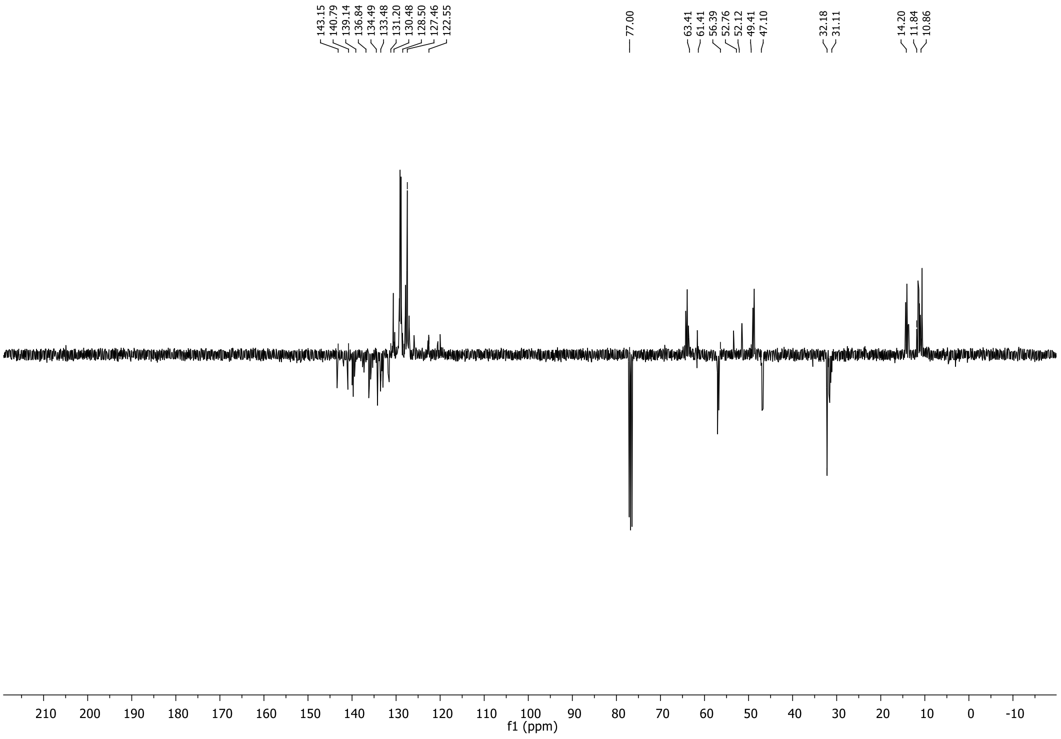

Figure S5. ^{1313}C-NMR of **2b** in $CDCl_3$.

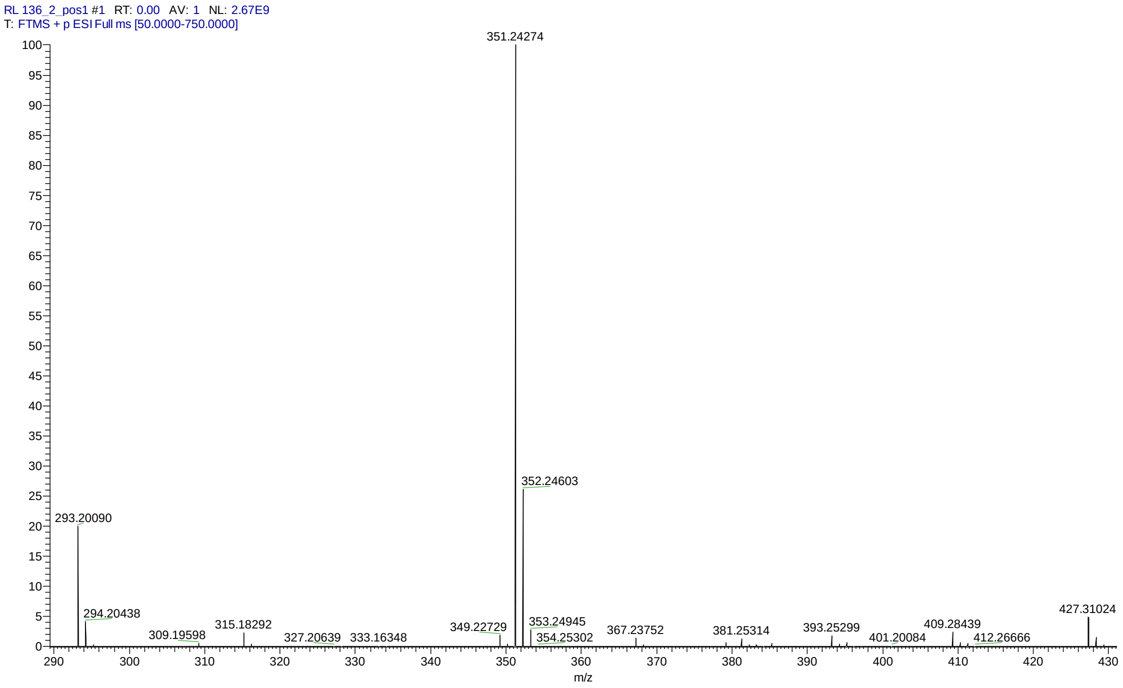

Figure S6. ESI mass spectrum of **2b** acquired in positive mode.

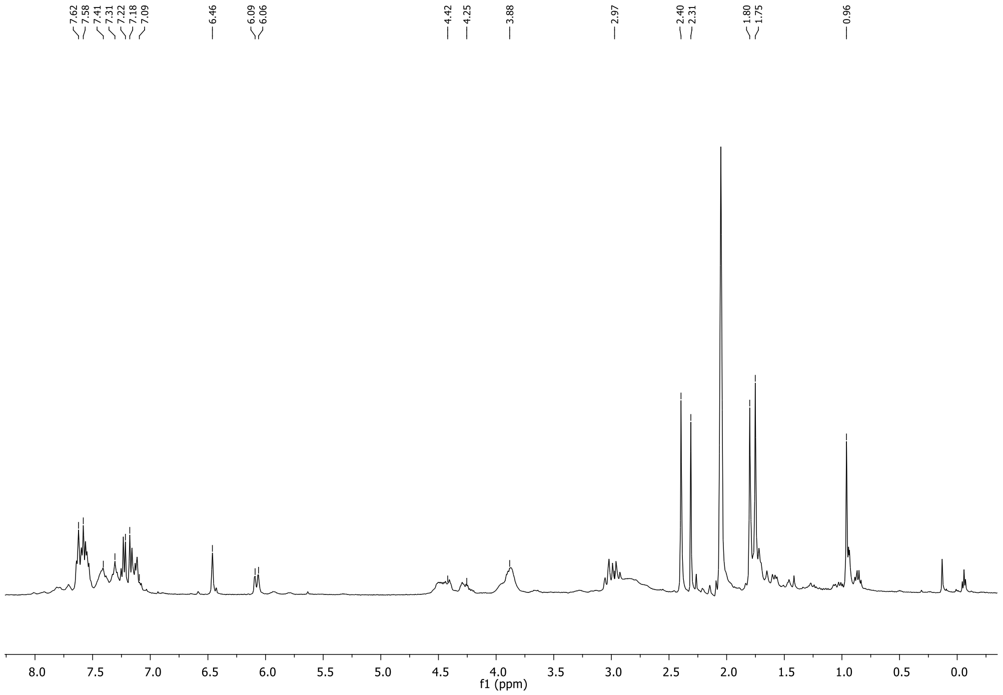

Figure S7. ^{1}H-NMR of **3a** in acetone-d_6.

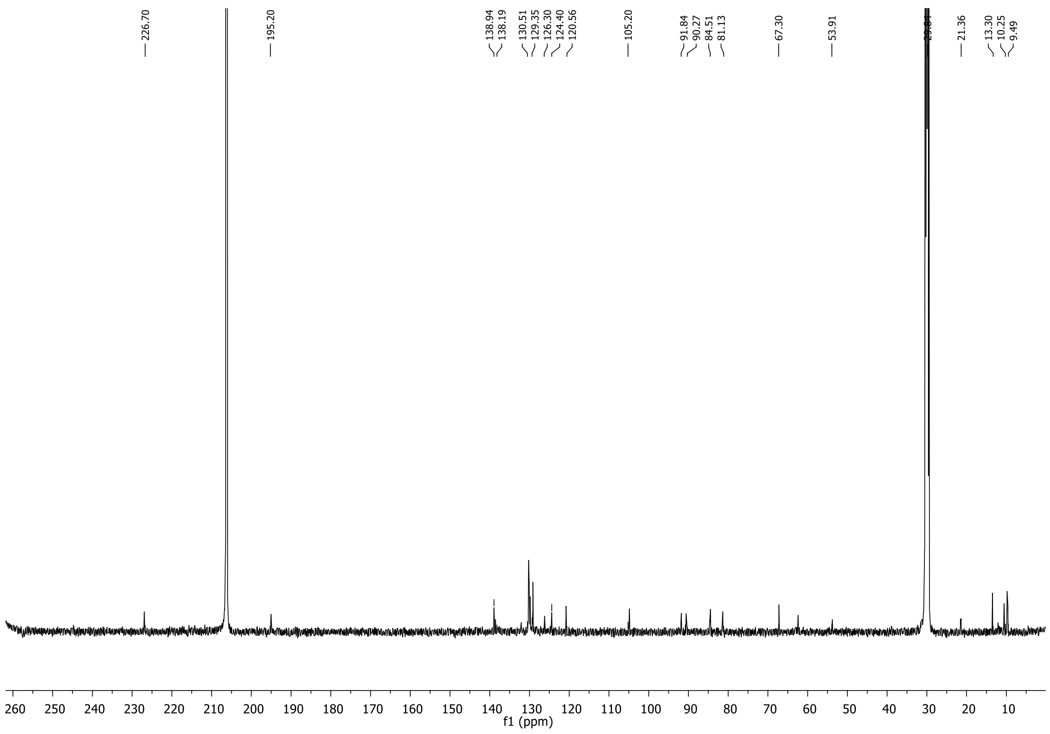

Figure S8. ^{13}C-NMR of **3a** in acetone-d_6.

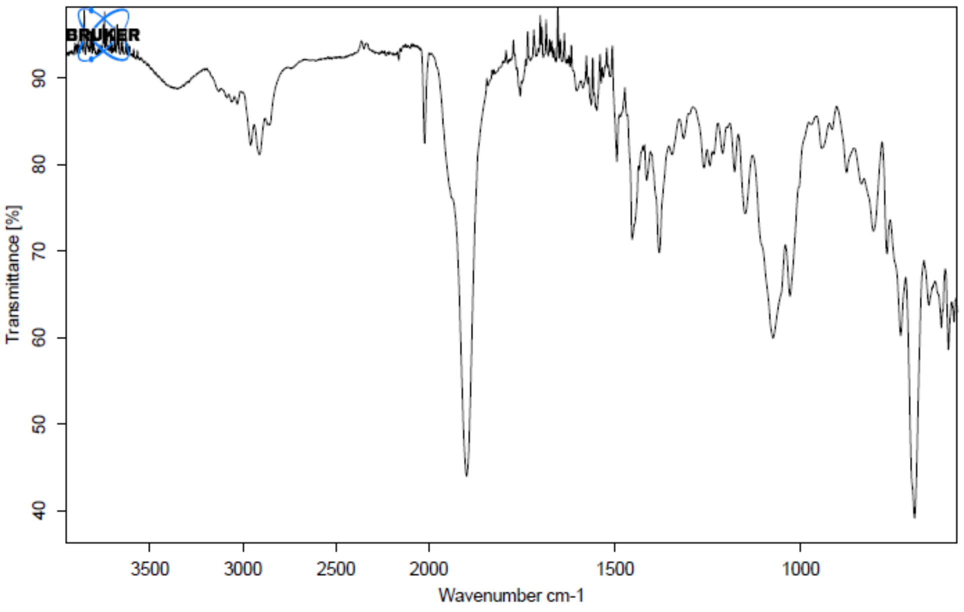

Figure S9. Infrared spectrum (KBr) of **3a.**

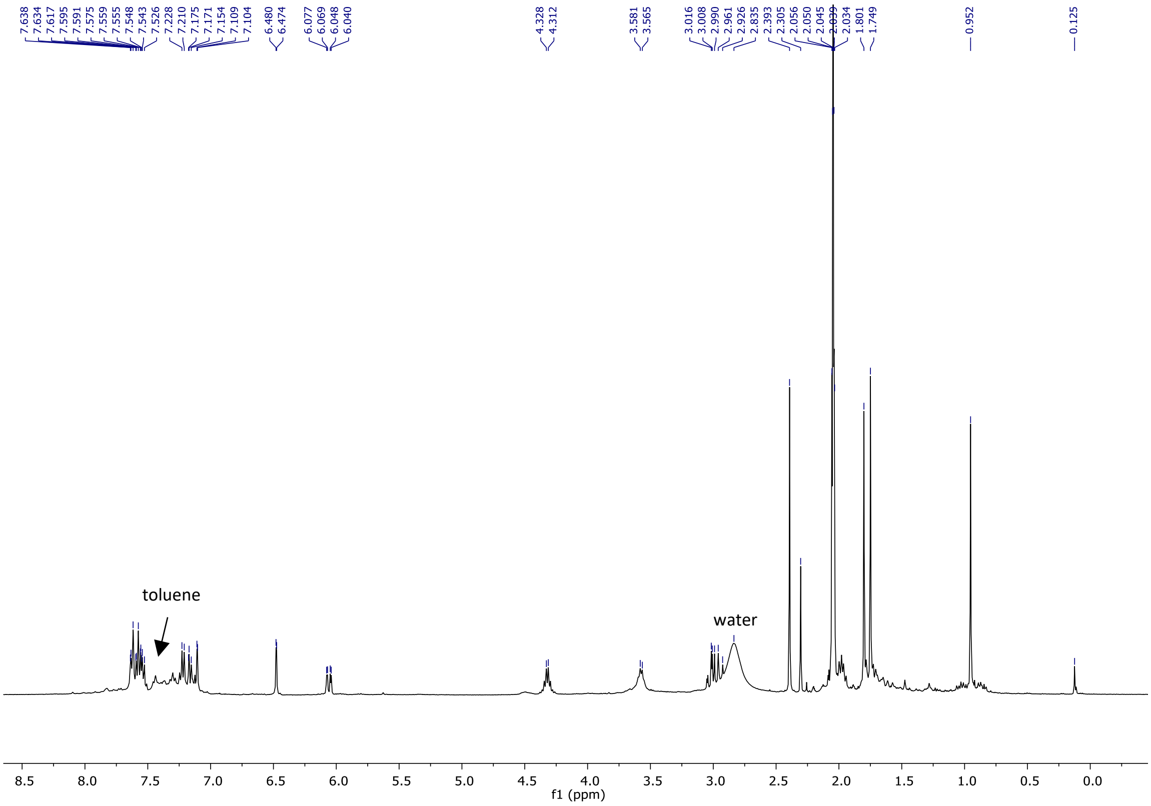

Figure S10. ^{1}H-NMR of **3b** in acetone-d_6.

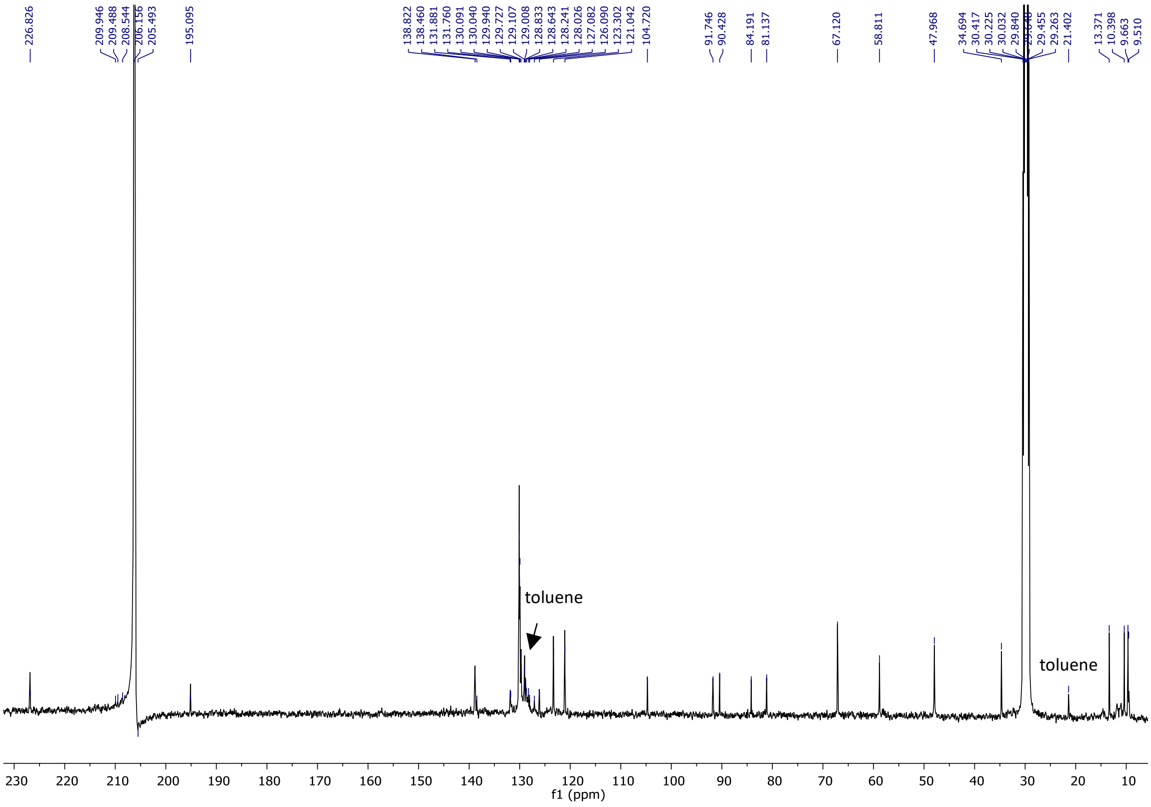

Figure S11. ^{13}C-NMR of **3b** in acetone-d_6.

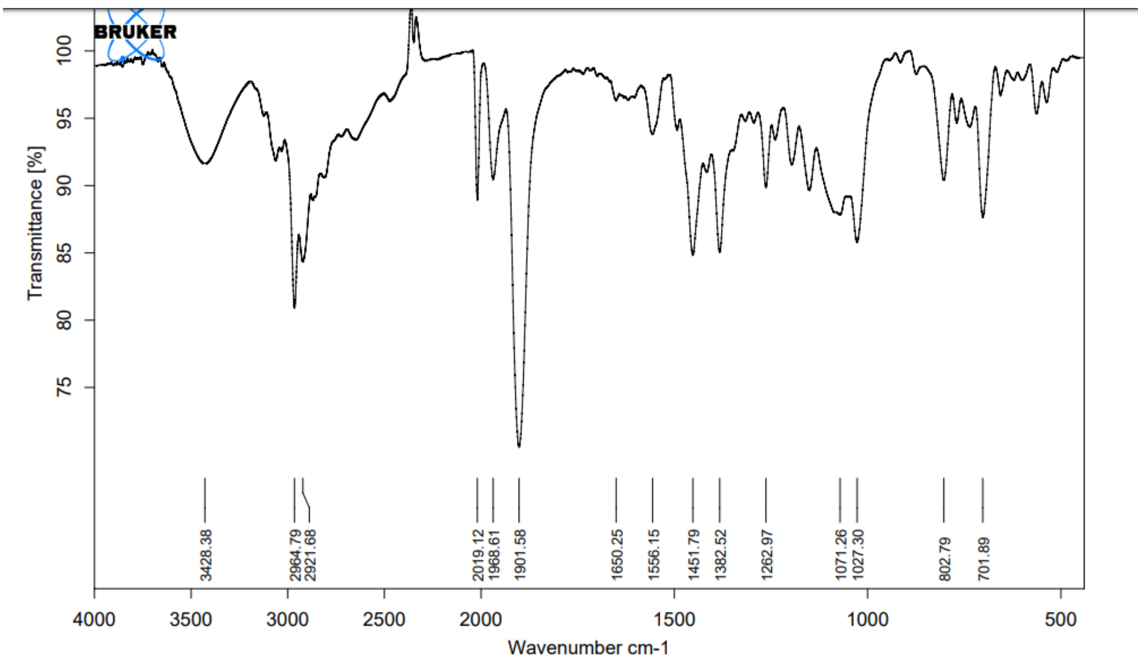

Figure S12. Infrared spectrum (KBr) of **3b.**

E Factor Calculation

E Factor = Total waste (kg) / Total product (kg)

For each case:

E Factor= [(mass of **1** + mass of iodoethane +mass of acetone)- mass of **2d** obtained]/ mass of **2d** obtained
